# Paratesticular dedifferentiated liposarcoma with leiomyosarcomatous differentiation: a case report with a review of literature

**DOI:** 10.1186/1746-1596-8-142

**Published:** 2013-08-23

**Authors:** Kazuhito Hatanaka, Takako Yoshioka, Takashi Tasaki, Akihide Tanimoto

**Affiliations:** 1 Department of Molecular and Cellular Pathology, Kagoshima University Graduate School of Medical and Dental Sciences, 8-35-1 Sakuragaoka, Kagoshima 890-8544, Japan

**Keywords:** Dedifferentiated liposarcoma, Leiomyosarcomatous differentiation, Paratesticular region

## Abstract

**Abstract:**

Paratesticular liposarcoma is a rare neoplasm, described in single case studies or components of larger studies, as histologically well-differentiated liposarcoma (WDL) and dedifferentiated liposarcoma (DL). However, leiomyosarcomatous differentiation is an extremely rare occurrence in WDL and DL. We report a case of leiomyosarcomatous differentiation in a 77-year-old man. The patient presented with a painless right scrotal mass. Magnetic resonance imaging showed a large mass along the right spermatic cord. The resected mass, measuring 17.5 × 12 × 5 cm, was composed of a high-grade pleomorphic undifferentiated sarcomatous component with necrosis. Atypical smooth muscle differentiation was also detected. Additional tumor sampling revealed the presence of a WDL component. Immunohistochemical analysis of the pleomorphic sarcomatous component showed positive staining for MDM2 and CDK4, and negative staining for alpha smooth muscle actin (αSMA) and desmin. The smooth muscle component was positive for αSMA and desmin, and negative for MDM2 and CDK4. Extension from primary retroperitoneal sarcoma was not proved. We diagnosed of DL with leiomyosarcomatous differentiation.

**Virtual slides:**

The virtual slide(s) for this article can be found here: http://www.diagnosticpathology.diagnomx.eu/vs/1484291498104021.

## Background

Liposarcoma is the most common type of soft-tissue sarcoma, accounting for about 20% of all mesenchymal malignant tumors. It usually arises from the thigh and retroperitoneum. However, pleomorphic liposarcomas arising in the foot and ankle have been reported [1]. The occurrence of liposarcomas in the paratesticular regions is also rare [2]. A study of 30 cases of paratesticular liposarcoma showed that 19 cases were well-differentiated liposarcoma (WDL), 10 cases were dedifferentiated liposarcoma (DL), and 1 showed a myxoid/round cell liposarcoma [2]. DL is usually composed of atypical lipomatous tumor (ALT)/WDL areas and dedifferentiated components that usually overlap with spindle/pleomorphic cell high-grade sarcoma or myxoid/spindle cell low-grade sarcoma. Dedifferentiated areas rarely show heterologous differentiation with myogenic, osteo/chondrosarcomatous, or angiosarcomatous elements [3]. Moreover, the peculiar meningothelial-like whorling and metaplastic bone formation were reported as other elements of heterologous differentiation [4]. Leiomyosarcoma, rhabdomyosarcoma, chondrosarcoma, and osteosarcoma differentiation has also been reported in malignant mesenchymal tumor [5].

Paratesticular liposarcoma with leiomyosarcomatous differentiation is extremely rare; to our knowledge, only single case studies or components of larger studies have been reported in the English literature [6-9]. Here, we report a very rare case of paratesticular DL with leiomyosarcomatous differentiation with a review of literature.

## Case presentation

A 77-year-old man with diabetes mellitus presented with a growing mass in the right testis of 3 months duration. A computed tomographic scan revealed a soft tissue mass, measuring 12 cm in diameter, in the right scrotal sac. The mass showed low signal intensities on T2-weighted magnetic resonance imaging. Radical inguinal orchiectomy was performed because of suspected malignancy.

## Materials and methods

The resected specimens were fixed with 10% neutral-buffered formalin and embedded in paraffin blocks. Four micrometer-thick paraffin sections were cut and mounted on glass slides, deparaffinized in xylene, rehydrated with ethanol, and immunostained with the following antibodies: MDM2 (monoclonal, 1B10, Novocastra, Newcastle upon Tyne, UK), CDK4 (monoclonal, DCS-31, Invitrogen, Camarillo, CA), S-100 protein (polyclonal, Z0311, Dako, Glostrup, Denmark), alpha smooth muscle actin (αSMA) (monoclonal, αsm-1, Novocastra), desmin (monoclonal, D33, Dako, Glostrup, Denmark), and MyoD1 (monoclonal, 5.8A, Dako, Glostrup, Denmark). Sections were stained using Dako Envision kit (Dako, Glostrup, Denmark).

## Results

**Figure 1:**
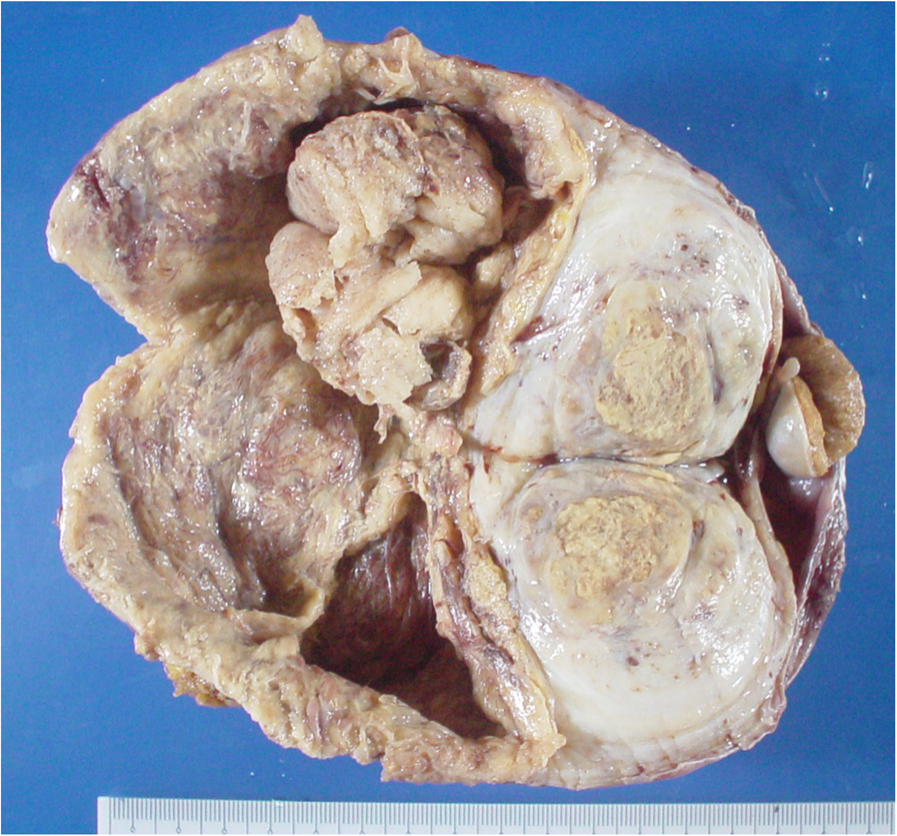
**Macroscopic tumor characteristics.** Nodular mass showing a white-yellow to red-brown cut surfaces with marked necrosis.

**Figure 2:**
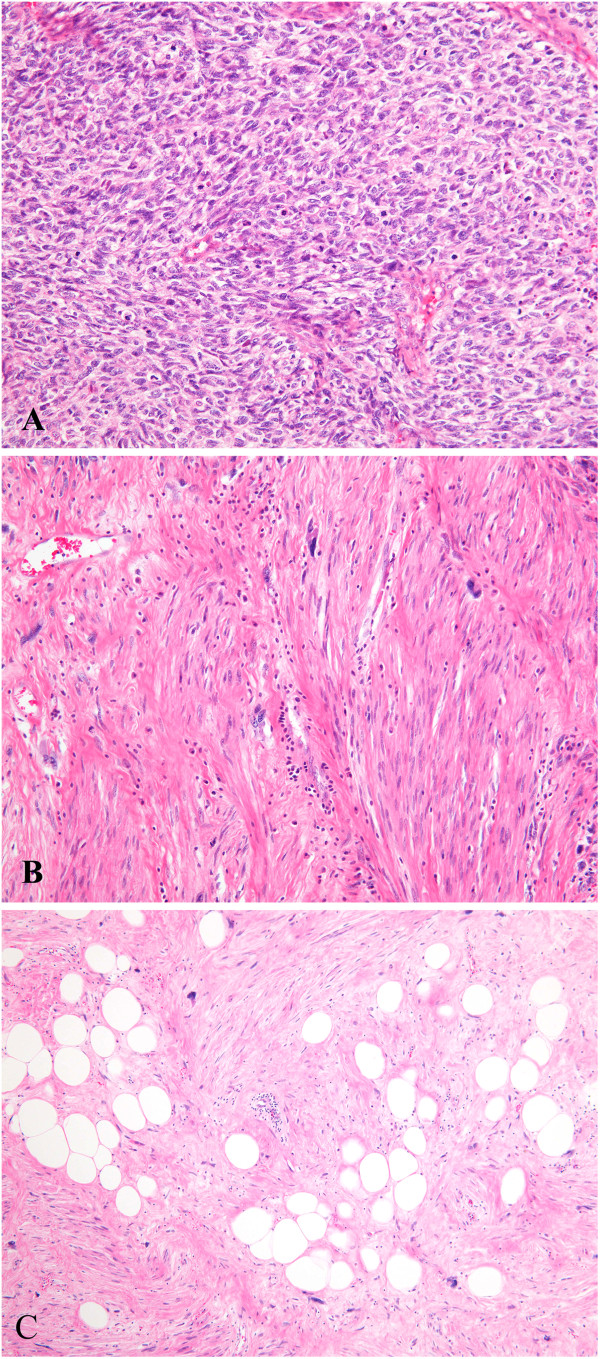
**Microscopic tumor characteristics. (A)** Proliferation of atypical short spindle or oval cells resembling high grade undifferentiated sarcoma. **(B)** Proliferating spindle cells containing elongated, blunt-ended nuclei and eosinophilic fibrillar cytoplasm with scattered enlarged and irregular nuclei. **(C)** The presence of a WDL component, showing mature-appearing adipose tissue and fibrous bands with irregular nuclei.

On macroscopic examination, a nodular mass measured 17.5 × 12 × 5 cm, and showed a white-yellow to red-brown cut surface with marked necrosis (Figure 1). The distribution of adipose tissue was unclear in the tumor. Microscopic examination revealed that the tumor was composed mainly of proliferating atypical short spindle or oval cells that resembled high-grade undifferentiated sarcoma (Figure 2A). The cells showed relatively high mitotic activity with more than 5 mitoses per 10 high-power fields. Necrosis and hemorrhage were also present, and necrosis was observed in approximately 40% of the tumor. Moreover, fascicles of spindle cells containing elongated, blunt-ended nuclei and eosinophilic fibrillar cytoplasm with scattered enlarged and irregular nuclei were also detected (Figure 2B). Osteo/chondrosarcomatous differentiation and meningothelial-like whoring pattern were not observed. The presence of the WDL component was proved in additional samples from the tumor (Figure 2C).

**Figure 3:**
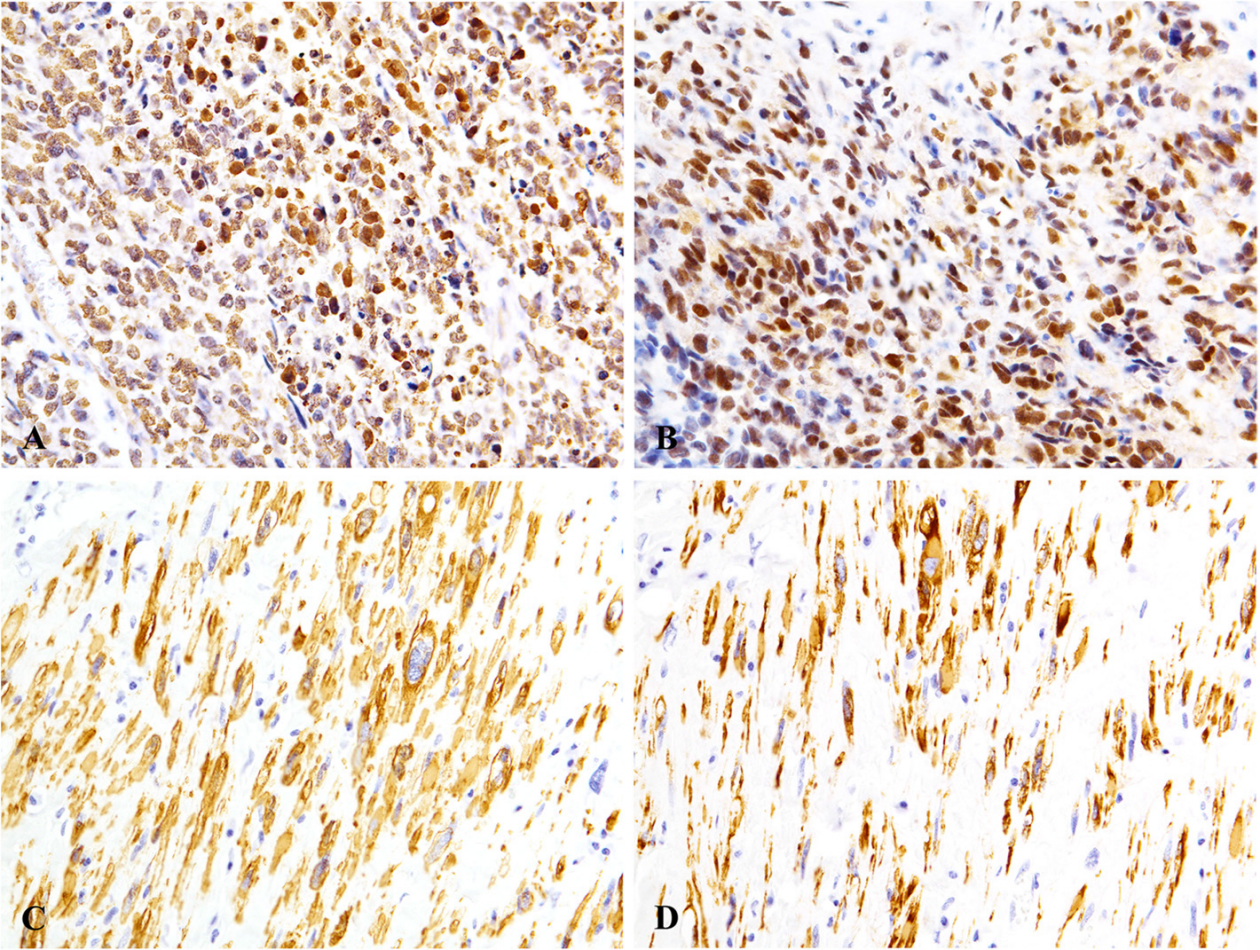
**Immunohistochemical analysis. (A)(B)** Sarcomatous component is positive for MDM2 **(A)** and CDK4 **(B). (C)(D)** Spindle cells are positive for alpha smooth muscle actin **(C)** and desmin **(D)**.

On immunohistochemical staining, the high-grade sarcomatous component was positive for MDM2 (Figure 3A) and CDK4 (Figure 3B), but not for S-100 protein, αSMA, and desmin. The spindle cell component was positive for αSMA (Figure 3C) and desmin (Figure 3D), but not for MDM2 and CDK4. Both components showed no reactivity to MyoD1. The resected margin was free, and extension from primary retroperitoneal sarcomas was not proved.

DL with leiomyosarcomatous differentiation was diagnosed. The leiomyosarcomatous component occupied approximately 10% of the tumor. The patient was disease free after at the 2-year follow-up.

## Discussion

Paratesticular liposarcoma is rare and has been reported in single case reports or as components of larger series. These tumors are thought to arise de novo from the adipose tissue around the spermatic cord or by malignant transformation of pre-existing lipomas [10]. In 2003, Montogomery et al studied 30 cases of paratesticular liposarcomas in men aged 41 – 87 years (mean age, 63 years) [2]. The tumors involved the spermatic cord (23 cases), testicular tunics (6 cases), or epididymis (1 case) and measured from 3 - 30 cm (mean, 11.7 cm). From the histological viewpoint, 19 cases were WDL, 10 were DL, and 1 was a myxoid/round cell liposarcoma. Among 10 DL cases, 5 were high-grade and 5 were low-grade. Leiomyosarcomatous differentiation was not described in this series. Binh et al reported 27 cases of DL with divergent myosarcomatous differentiation, including 7 cases in the paratesticular area, and leiomyosarcomatous differentiation was detected in 7 cases [9]. Henricks et al reported 155 cases of DL, including 13 cases of spermatic cord/scrotum origin, and leiomyosarcomatous differentiation was observed in 2 of the 155 cases [11]. However, the relationship between the tumor location and the histological findings was not available in both studies.

To the best of our knowledge, only 3 well-documented cases of paratesticular liposarcoma with leiomyosarcomatous differentiation have been reported in the English literature [6-8]. The clinicopathological features of the reported cases, including the present case, are summarized in Table 1. In case 1, the tumor measured 7 × 3.5 × 3 cm, and was composed of ALT/WDL with well-differentiated leiomyosarcomatous component. The smooth muscle cells were arranged in well-defined bundles with no prominent nuclear pleomorphism or mitosis. Immunostaining was not performed. In case 2, the tumor measured 25 × 21 × 8 cm, and was composed of a WDL component with moderate leiomyosarcomatous differentiation. Proliferation of spindle cells, with enlarged, pleomorphic, and hyperchromatic nuclei were observed arranged in fascicles, with focal hemorrhage and necrosis in the leiomyosarcomatous area. On immunohistochemical examination, the leiomyosarcomatous components were positive for αSMA, desmin, and vimentin and negative for S-100 protein, keratin, CAM 5.2, and factor VIII-related antigen. In case 3, the tumor measured 2 cm and was composed of WDL component with leiomyosarcomatous differentiation. The smooth muscle component showed low cellularity and low nuclear grade. Immunohistochemical evaluation was not available. In the present case, the tumor was composed of a pleomorphic sarcomatous component with leiomyosarcomatous differentiation. Liposarcoma with smooth muscle differentiation was described relatively recently, and consists of 2 types: WDL having foci of mature but histologically atypical smooth muscle tissue (so-called lipoleiomyosarcoma) and DL with smooth muscle differentiation in the dedifferentiated areas (DL with heterogeneous differentiation) [8]. According to this classification, cases 1, 2, and 3 are lipoleiomyosarcoma, and the present case is DL with heterogeneous differentiation.

**Table 1:** Clinicopathological features of well-documented cases of paratesticular liposarcoma with leiomyosarcomatous differentiation

**Case**	**Age (y)**	**Size (cm)**	**Classification of liposarcoma**	**Smooth muscle component**	**Recurrences**	**Metastases**	**Status**	**Interval (mo)**	**Reference**
1	49	7×3.5×3	ALT/WDL	Well-differentiated	No	NA	NED	30	[6]
2	70	25×21×8	WDL	Moderately differentiated	No	No	NED	72	[7]
3	65	2	WDL	Well-differentiated	Yes	No	NED	96	[8]
Current case	77	17.5×12.5×5	DL	Well-differentiated	No	No	NED	24	

*ALT* atypical lipomatous tumor, *WDL* well-differentiated liposarcoma, *DL* dedifferentiated liposarcoma, *NED* no evidence of disease.

It is important to recognize the coexistence of the ALT/WDL components in order to obtain a definitive diagnosis of DL. However, if these components are not recognized, the differential diagnosis includes a variety of lesions, benign or low-grade mesenchymal tumors for low-grade differentiation, and pleomorphic sarcomas for high-grade differentiation. The general differential diagnosis of adult spindle cell tumors in this site includes primarily fibrous mesothelioma, leiomyosarcoma, malignant fibrous histiocytoma, various benign fibrous tumors, pseudotumors, and fibromatosis [2]. In addition to morphological findings, immunohistochemical study, including MDM2 and CDK4 staining, may be useful for diagnosis. The main karyotypic alteration of DL is considered to be the presence of supernumerary ring chromosomes and/or giant chromosomes composed of a chromosome 12q13-15 sequence involving *MDM2* and often *CDK4* genes [12]. The utility of MDM2 and CDK4 immunostaining is well documented in the diagnosis of WDL and DL [13]. However, MDM2 and CDK4 are not specific markers for liposarcoma. In the present case, the leiomyosarcomatous component showed no immunopositivity for MDM2 and CDK4; however, some leiomyosarcomas show positivity for MDM2 and CDK4 [3]. Therefore, it is important to prove the existence of the ALT/WDL component through additional sampling of the tumor.

Clinically, the risk of local recurrence is not affected by myosarcomatous differentiation in DL, although the metastatic rate is relatively low compared to that of leiomyosarcoma. The prognosis of patients with DL with myosarcomatous differentiation is better than that of patients with leiomyosarcoma [9]. Therefore, from a clinical perspective, it is important to distinguish DL with myosarcomatous differentiation from leiomyosarcoma in an argument for conventional adjuvant chemotherapy.

In summary, we report a rare case of paratesticular DL with with leiomyosarcomatous differentiation in a 77-year-old man. This case highlights the importance of additional sampling for obtaining a definitive diagnosis of DL, especially if the existence of an ALT/WDL component is not noted.

## Consent

Written informed consent was obtained from the patient for publication of this case report and any accompanying images. A copy of the written consent is available for review by the Editor-in-Chief of this journal.

## Competing interests

All authors declare that they have no competing interests.

## Authors’ contributions

KH and AT participated in conception of the idea and writing of the manuscript. TY and TT performed the histopathological interpretation of the tumor tissue. All authors read and approved the final manuscript.

## References

[B1] Brcić L Jakovcević A Vuletić LB Orlić D Seiwerth S Pleomorphic liposarcoma of the foot: a case report Diagn Pathol 2008 3 15 10.1186/1746-1596-3-15 18416844 PMC2358876

[B2] Montgomery E Fisher C Paratesticular liposarcoma: a clinicopathologic study Am J Surg Pathol 2003 27 40 47 10.1097/00000478-200301000-00005 12502926

[B3] Dei Tos AP Liposarcoma: new entities and evolving concepts Ann Diagn Pathol 2000 4 252 266 10.1053/adpa.2000.8133 10982304

[B4] Song JS Gardner JM Tarrant WP Shen S Ayala AG Yu E Ro JY Dedifferentiated liposarcoma with peculiar meningothelial-like whorling and metaplastic bone formation Ann Diagn Pathol 2009 13 278 284 10.1016/j.anndiagpath.2008.07.002 19608088

[B5] Li YF Yu CP Wu ST Dai MS Lee HS Malignant mesenchymal tumor with leiomyosarcoma, rhabdomyosarcoma, chondrosarcoma, and osteosarcoma differentiation: case report and literature review Diagn Pathol 2011 6 35 10.1186/1746-1596-6-35 21496258 PMC3094258

[B6] Evans HL Smooth muscle in atypical lipomatous tumors Am J Surg Pathol 1990 14 714 718 10.1097/00000478-199008000-00002 2378392

[B7] Suster S Wong TY Moran CA Sarcomas with combined features of liposarcoma and leiomyosarcoma: study of two cases of an unusual soft-tissue tumor showing dual lineage differentiation Am J Surg Pathol 1993 17 905 911 10.1097/00000478-199309000-00006 8352375

[B8] Flope AL Weiss SW Lipoleiomyosarcoma (well-differentiated liposarcoma with leiomyosarcomatous differentiation): a clinicopathologic study of nine cases including one with dedifferentiation Am J Surg Pathol 2002 26 742 749 10.1097/00000478-200206000-00007 12023578

[B9] Binh MB Guillou L Hostein I Château MC Collin F Aurias A Binh BN Stoeckle E Coindre JM Dedifferentiated liposarcomas with divergent myosarcomatous differentiation developed in the internal trunk: a study of 27 cases and comparison to conventional dedifferentiated liposarcomas and leiomyosarcomas Am J Surg Pathol 2007 31 1557 1566 10.1097/PAS.0b013e31804b4109 17895758

[B10] Khoubehi B Mishra V Ali M Motiwala H Karim O Adult paratesticular tumours BJU Int 2002 90 707 715 10.1046/j.1464-410X.2002.02992.x 12410753

[B11] Henricks WH Chu YC Goldblum JR Weiss SW Dedifferentiated liposarcoma: a clinicopathological analysis of 155 cases with a proposal for an expanded definition of dedifferentiation Am J Surg Pathol 1997 21 271 281 10.1097/00000478-199703000-00002 9060596

[B12] Pedeutour F Forus A Coindre JM Berner JM Nicolo G Michiels JF Terrier P Ranchere-Vince D Collin F Myklebost O Turc-Carel C **Structure of the supernumerary ring and giant rod chromosomes in adipose tissue tumors**. Genes chromosomes Cancer 1999 24 30 41 9892106

[B13] Binh MB Sastre-Garau X Guillou L de Pinieux G Terrier P Lagacé R Aurias A Hostein I Coindre JM MDM2 and CDK4 immunostainings are useful adjuncts in diagnosing well-differentiated and dedifferentiated liposarcoma subtypes: a comparative analysis of 559 soft tissue neoplasms with genetic data Am J Surg Pathol 2005 29 1340 1347 10.1097/01.pas.0000170343.09562.39 16160477

